# Biogeography pattern of the marine angiosperm *Cymodocea nodosa* in the eastern Mediterranean Sea related to the quaternary climatic changes

**DOI:** 10.1002/ece3.8911

**Published:** 2022-05-25

**Authors:** Ioannis Konstantinidis, Georgios A. Gkafas, Vasillis Papathanasiou, Sotiris Orfanidis, Frithjof C. Küpper, Sophie Arnaud‐Haond, Athanasios Exadactylos

**Affiliations:** ^1^ Genomics Division Faculty of Biosciences and Aquaculture Nord University Bodø Norway; ^2^ Department of Ichthyology and Aquatic Environment School of Agricultural Sciences University of Thessaly Volos Greece; ^3^ Fisheries Research Institute (HAO DEMETER) Kavala Greece; ^4^ School of Biological Sciences University of Aberdeen Aberdeen UK; ^5^ Department of Chemistry Marine Biodiscovery Centre University of Aberdeen Aberdeen UK; ^6^ Ifremer UMR MARBEC (Marine Biodiversity, Exploitation and Conservation) Sète Cedex France

**Keywords:** *Cymodocea nodosa*, Holocene, Mediterranean Sea, migration, Pleistocene, population structure, sea‐level rise

## Abstract

We investigated the population dynamics of a highly clonal marine angiosperm, *Cymodocea nodosa*, in the eastern Mediterranean Sea, to identify the historical dynamics, demography, and connectivity of the species in the area. Eighteen microsatellite loci were used in conjunction with coalescent methods to investigate the genetic structure and demographic history of *C. nodosa* meadows. Approximate Bayesian computation (ABC) modeling was used to examine the pattern of divergence over time in the context of environmental change over the course of the Quaternary period. ABC analysis revealed an initial split of the *C. nodosa* populations between the north‐western, northern, and north‐eastern Aegean Sea during the Pleistocene epoch, followed by a more recent divergence of the north‐western population and the central‐western part of the Aegean Sea. According to the results, the most parsimonious historical scenario is that of a pervasive genetic signature of the effects of the drop in sea level during the Pleistocene epoch. This scenario supports the isolation of the north‐western, north, and north‐eastern area, and the subsequent recolonization after post‐glaciation sea level rise that may explain the north‐western differentiation as well present‐day detected dispersion of *C. nodosa*.

## Graphical abstract

We investigated the population dynamics of a highly clonal marine angiosperm, *Cymodocea nodosa*, in the eastern Mediterranean Sea, to identify historical dynamics, demography, and connectivity of the species in the area. The most parsimonious historical scenario is that of a pervasive genetic signature of the effects of the drop of Aegean Sea level during the Pleistocene epoch. This scenario supports the isolation of the north‐western, north, and north‐eastern area, and the subsequent recolonization after post‐glaciation Aegean Sea level rise that may explain the north‐western differentiation as well present day detected dispersion of *C. nodosa*.

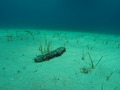

## 1 INTRODUCTION

The limited knowledge of the nature of boundaries in the marine environment limits the understanding of the way the genetic structure is shaped in the oceans. However, studying past environmental conditions such as the glaciation cycles during the Quaternary period can provide us with hints of the current population structure of many marine organisms (Arnaud‐Haond et al., 2007; Chefaoui et al., 2017; Hofreiter & Stewart, 2009). The Mediterranean Sea is a key region for studying the effects and consequences of the last glacial maximum (LGM). During this period, major changes in global sea level were observed due to water mass exchange between ice caps and oceans (Lambeck & Chappell, 2001). In addition, oceanic and atmospheric patterns from the North Atlantic as well as climatic phenomena linked to the African and Asian monsoons have been shaping the environmental factors of the Mediterranean basin (Alpert et al., 2006). Vicariance between populations of many terrestrial and marine organisms is the result of Pleistocene glaciation events (Hewitt, 1999, 2000). Rohling's work (as cited in Hewitt, 2000) suggests that during major glaciations the accumulation of ice on mountainous regions could reduce the global sea level by 120 m. Furthermore, Collina‐Girard (2001) reports that during the last glacial maximum (at about 11 kyrBP), the Mediterranean basin was divided into two major sub‐basins due to sea‐level changes (−120/130 m) and the connectivity of the eastern and western basin was significantly limited (Thiede, 1978). That being said, major changes in species movement, distribution, genetic diversity, and heterogeneity are expected in response to climate changes (Hewitt, 1999) including sea‐level rise (Küpper & Kamenos, 2018).

At the end of the Pleistocene epoch, differences have been observed between the two isolated basins as a response to the climatic and hydrological processes. While anoxic conditions occurred in the east due to the sapropel S1 formation (9500–6000 years before present), the western basin was affected mainly by the lower export production fluxes, suggesting that different processes have resulted in contrasting conditions in each basin (Martínez‐Ruiz et al., 2003). Allen et al. (1999) report that the western and central Mediterranean were influenced by the Northern hemisphere climate and that they were linked with the Atlantic climate system during the last glacial period. The climatic fluctuations and connectivity status of the Mediterranean basin during this period might have resulted in a pervasive modification of biotic and abiotic factors shaping the distribution of diversity over the last millennia. Ludt and Rocha (2015) stress the importance of sea‐level change as it can play a significant role in the population differentiation and genetic structure of many marine taxa.

During the last glacial maximum, a temperature shift at the entrance of the Mediterranean, combined with lower temperature in the western than in the eastern Mediterranean basin (Thiede, 1978), may have been of major importance for the development of temperature ecotypes and geographic distribution of lineages in this region (Orfanidis & Breeman, 1999; and literature within). This east–west disconnection and environmental heterogeneity, mainly during the transition from Pleistocene to Holocene and the post‐glacial era, have been proposed to explain the present‐day patterns of genetic differentiation for several other organisms such as mollusks (Giantsis et al., 2019; Ladoukakis et al., 2002; Nikula & Väinölä, 2003), echinodermata (Konstantinidis et al., 2017; Valente et al., 2015), crustaceans (Ragionieri & Schubart, 2013; Zane et al., 2000), fish (Bahri‐Sfar et al., 2000; Exadactylos et al., 2019; Gkafas et al., 2013; Kousteni et al., 2015; Sanna et al., 2013; Wilson & Eigenmann Veraguth, 2010; Woodall et al., 2011, 2015), and mammals (Gaspari et al., 2015; Gkafas et al., 2017; Moura et al., 2014).

The population structure of seagrasses has also supported this hypothesis of a strong influence of the Quaternary period. Arnaud‐Haond et al. (2007) indicated that the last ice age potentially shaped the biogeographical distribution of *Posidonia oceanica*, demonstrating minimum gene flow among seagrass meadows between the eastern and western Mediterranean and supporting a low level of migration since Pleistocene recolonizations (Arnaud‐Haond et al., 2014). In addition, Serra et al. (2010) and Procaccini et al. (2001) showed that present environmental conditions may also contribute to explaining the maintenance of the east–west cleavage between the Mediterranean *P. oceanica* populations, that have restricted gene flow among them. Alberto et al. (2008) have shown that *Cymodocea nodosa* populations have very strong genetic structure throughout the Mediterranean basin, as well as significantly distinct populations at the edges of their distribution (east Mediterranean—south‐east Atlantic).

Even though seagrass population structures appear to be significantly different between the two Mediterranean basins, there is still a paucity of data for many species including seagrasses, from the eastern Mediterranean basin. Studies have shown that several populations of the angiosperm *C. nodosa* within the eastern Mediterranean waters are closely related (Alberto et al., 2008). During the Pleistocene, it is plausible that populations of *C. nodosa*, which is well adapted to the temperate climate and warm conditions (Procaccini et al., 2007; Tsioli et al., 2019), had to retreat from the northern Mediterranean coasts toward the southern edges of the species distribution, where most of the glacial refugia have been reported (Alberto et al., 2008; Orfanidis & Breeman, 1999; Ruggiero et al., 2004). At a later time point (post‐Pleistocene), these refugia appear to have been the source of re‐colonization for the eastern Mediterranean basin, which may explain the close genetic relationship among geographically distant meadows observed in this area.

The Aegean Sea is a key region that encompasses contrasting biotic and abiotic characteristics, especially for seagrasses (Exadactylos, 2015; Malandrakis et al., 2017). The two major basins (north and south Aegean) are separated by the Cyclades plateau which plays the role of a physical barrier with a maximum depth of 400 m. Studies on the seagrass *C. nodosa* report genetic differentiation of the species within the north Aegean Sea (Gkafas et al., 2016) particularly in isolated gulfs by cyclonic and anti‐cyclonic features (Petihakis et al., 2002). The geomorphology of the area as a whole is characterized by a heterogeneous assemblage of sandy beaches and rocky shores, brackish lagoons and river delta systems, deep basins, and shallow shelves (Karageorgis et al., 2005). All these ecosystemic features may contribute to the limitation of gene flow and may thus play a significant role in the population structure of many species. Studies on benthic species such as sponges (Kefalas et al., 2003; Voultsiadou, 2005), crustaceans (Exadactylos, 2008; Gkafas et al., 2019), and anthozoa (Vafidis, 2002) confirm differentiation of several marine species in that area. These studies suggest a role of a diversity of factors including geographical distance (north‐central‐south Aegean), geomorphological characteristics (benthic substrates), and physicochemical features such as the oligotrophic high‐salinity warm water inflow from the south (Levantine Sea) and the nutrient‐rich cold brackish water inflow from the Black Sea and northern rivers (Lykousis et al., 2002; Voultsiadou et al., 2008; Zervakis et al., 2004).

Following our recent studies of transcriptomic responses (Malandrakis et al., 2017) as well as photosynthetic performance and growth (Tsioli et al., 2019) in *C. nodosa* under extreme temperature, salinity, and their combination, but also the epiphytes of the leaves of this seagrass species (Tsioli et al., 2021), this study was conducted to test the hypothesis of the influence of Pleistocene sea‐level changes and Holocene warming, as well as of the present day on environmental heterogeneity on the distribution of genetic polymorphism of *C. nodosa* across the Greek coastline from north to south, having in mind the already described genetic structure pattern in the Aegean Sea. To test this hypothesis, a portfolio of 335 *C. nodosa* samples from 12 different geographical meadows were analyzed with 18 microsatellite data, to assess the most likely demographic scenario using the approximate Bayesian computational (ABC).

## 2 MATERIAL AND METHODS

### 2.1 Samples collection and study area

**FIGURE 1:**
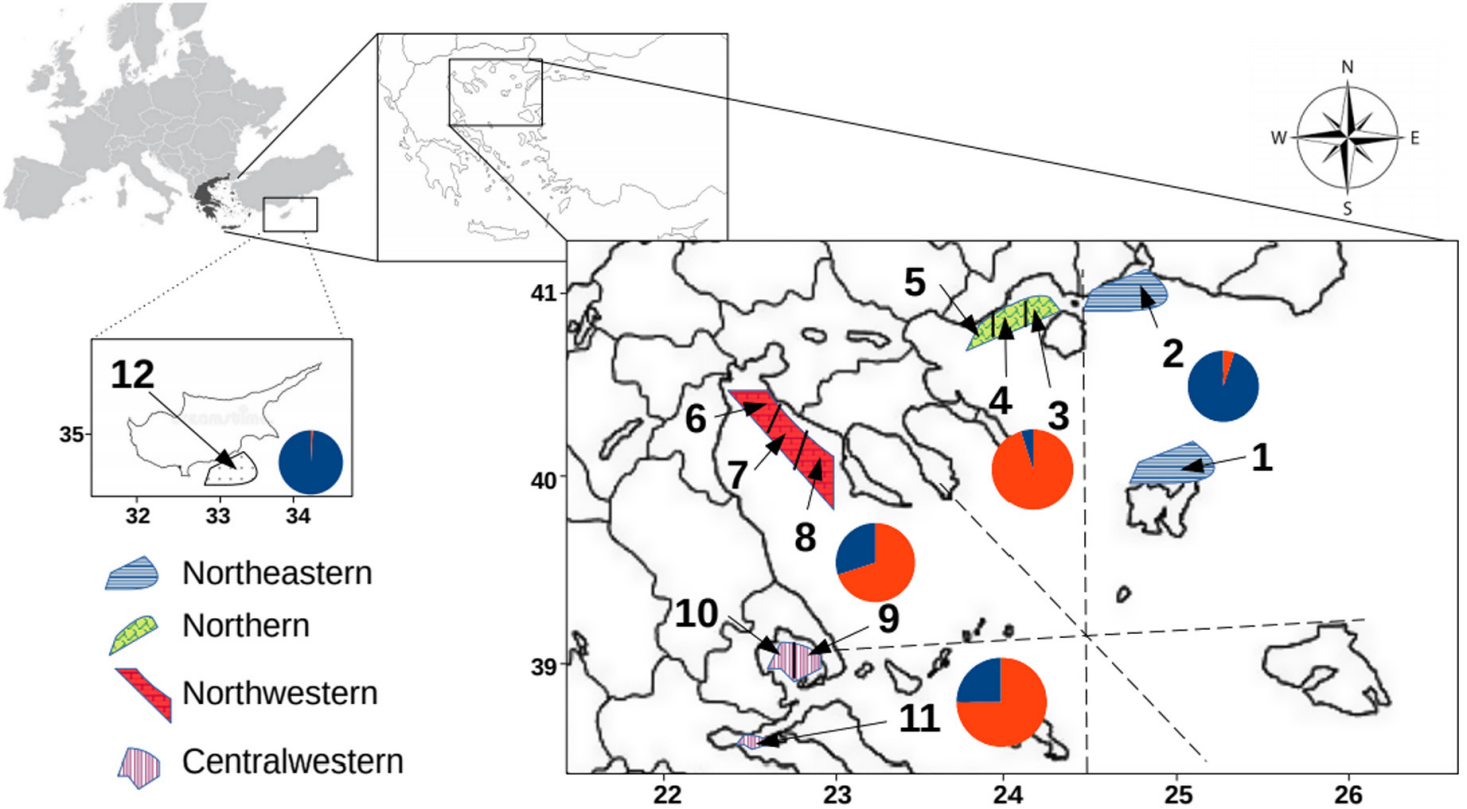
Sampling sites of *Cymodocea nodosa* meadows; 1: Lemnos (8), 2: Imeros (32), 3: Fanari (34), 4: Vrasidas (34), 5: Nea Karvali (22). 6: Viamyl (23), 7: Ag. Triada (38), 8: Chalkidiki (9), 9: eastern Pagasitikos Gulf (47), 10: western Pagasitikos Gulf (36), 11: Maliakos Gulf (37), 12: Cyprus (11). Numbers in parentheses indicate the number of samples. The color pattern illustrates blue for north‐eastern; green for northern; red for north‐western; purple for central‐western. Pies indicate the genetic clusters (K = 2) as having been implemented in STRUCTURE software

In total 12 meadows were sampled (Figure 1). Population 1, Lemnos meadow, is located on the central‐eastern coasts of Mudros Gulf in proximity to the Mudros village on the island of Lemnos in a shallow area with extensive seagrass bed and less anthropogenic impact. Imeros (population 2) and Fanari (population 3) are located on the western Thracian coasts and are considered Natura 2000 sites (code GR1130009), while extensive agriculture in the wider region has a potential negative impact on these seagrass meadows. Nea Karvali (population 4) is located on the eastern coast of the Kavala Gulf while Vrasidas (population 5) is on the western. Nea Karvali, one of the most impacted areas of the Gulf, is located close to a phosphorus fertilizer plant, wastewater treatment works, and a crude oil de‐sulphurization complex. Vrasidas meadow is in the inner part of Cape Vrasidas, Eleutheron Gulf, and is one of the least impacted sites of Kavala Gulf and also part of the Natura 2000 network (code GR1150009), that receives limited fishing and seasonal touristic activity. Populations 6, 7, and 8 consist of the north‐western cluster of populations and are located in Thermaikos Gulf. The Gulf is a shallow (max. depth 25 m) coastal water body in the northwest Aegean Sea. The area is considered to be one of the most polluted sites in Greece due to the high agricultural, urban, and industrial activity that surrounds Thessaloniki city, the second‐largest city in Greece. Viamyl meadow (population 6) is located in the north‐eastern part of the gulf, being subjected to local effluents from a small wastewater treatment plant, direct urban discharges, and freshwater inputs from the Athemundas stream. Agia Triada meadow (population 7) is located in Thermaikos Gulf, near a small community, 12 km west of Viamyl, with significant seasonal tourism. Chalkidiki meadow (population 8) is located in the far south part of Thermaikos Gulf, being subjected to high anthropogenic pressure mostly due to tourism. Populations 9 (eastern Pagasitikos) and 10 (western Pagasitikos) are located in the Pagasitikos Gulf and along with population 11, Maliakos Gulf, consist of the central‐western Aegean Sea cluster, with a rather large amount of anthropogenic pressure. In the case of Pagasitikos Gulf there is an almost stable dipole, an anticyclone in the east and a cyclone in the central‐western part, accompanied by smaller jets and eddies (Petihakis et al., 2002). Lastly, the meadow sampled in Cyprus (population 12) was located at the north‐eastern coast of Cape Greco, at Konnos beach, where the only anthropogenic impact is tourism‐related activity.

*Cymodocea nodosa* samples were collected by SCUBA or snorkeling from 1 to 3 m depth, following a hierarchical random design. Each meadow was divided into two stations 100 m apart, and from every station 15 sampling units (SUs: unique shoots or set of interconnected shoots) were collected haphazardly, at least 10 m apart from each other. Shoots were dissected and individually stored in dry silica gel until further analysis. In total 335 SUs were analyzed.

### 2.2 DNA extraction and PCR amplification

Shoots tissue samples were extracted following a standard phenol/chloroform extraction protocol (after Gkafas et al., 2016). A panel of 18 microsatellite DNA loci was tested (Table S1). A multiplex PCR Kit (Qiagen) with hot start Taq was used for the DNA amplification. The 18 pairs of primers were divided into four multiplex groups according to size range and florescent primer pigment. The PCR cycling profile was 95°C for 15 min; 30 cycles of 95°C for 1 min, annealing temperature for 30 s, and 72°C for 30 s; 72°C for 15 min. PCR products were verified by agarose gel electrophoresis. Amplified DNA products were screened on an ABI 3730 DNA Analyzer (Applied Biosystems) using the ROX500 size marker. Each specimen's alleles were scored by the software STRAND 2.0 (Toonen & Hughes, 2001) and 10% of genotypes were redone for error checking.

### 2.3 Within population genotypic and genetic parameters

The program RClone (Bailleul et al., 2016) through the R platform was used to check for the occurrence of Multi Locus Lineages (MLL) clustering Multi Locus Genotypes (MLGs) distinct by only a few mutations or scoring errors. Expected (H_EXP_) and observed heterozygosity (H_OBS_), along with the departure from Hardy–Weinberg through F_IS_ calculation using the formulations described by Weir and Cockerham (1984); significance tested using 10,000 permutations test were estimated using ARLEQUIN 3.5 (Excoffier & Lischer, 2010).

### 2.4 Population genetic differentiation

Fixation indexes (F_ST_) were estimated using the θ estimator of Weir and Cockerham (1984) and tested for significance using the 10,000 permutations test using ARLEQUIN 3.5 (Excoffier & Lischer, 2010). A factorial correspondence analysis (FCA with the “3D by populations”) was also computed using GENETIX 4.05.2 (Belkhir et al., 1996).

Population structure was further assessed through clustering analysis using STRUCTURE 2.3 (Pritchard et al., 2000) assuming correlated allele frequencies and admixture. Three independent repeats were run for each value of K (1 ≤ K ≤ 12). Following test runs, the burn‐in length and length of the simulation were set at 1,000,000 and 3,000,000 repetitions, respectively. STRUCTURE HARVESTER was used to assess the likelihood value of the different K values and to implement the ΔK method (Evanno et al., 2005) reflecting the highest hierarchical level of structuring (Earl & von Holdt, 2012).

BayesAss 3.0 (Wilson & Rannala, 2003) was used to infer the rates and directionality of contemporary migration. To achieve acceptance ratios between 20% and 60% values of allele frequency (DA), inbreeding (F) value (DF), and migration rate (DM) were set to 0.10, 0.30, and 0.50, respectively, using 3 × 10^6^ iterations and a burn‐in of 10^6^. The software MIGRATE‐n v. 3.2.6 (Beerli, 2006; Beerli & Felsenstein, 2001; Beerli & Palczewski, 2010) was used to infer‐rated and directionality of historical migration. Four chains used (1.0—1.4—3.0—1,000,000), values of recorded steps and burn‐in length were set to 5000 and 100,000, respectively. Mean priors' distribution for Theta and M were set both to 50 (delta value =25), using 1500 bins. To check for convergence for both Bayesian inference analyses, the software TRACER 1.6 was used (Rambaut et al., 2018). To visualize the connectivity between groups, we used the RCircos package (Zhang et al., 2013).

### 2.5 Biogeographic inferences

To compare demographic scenarios of divergence history, an ABC analysis was implemented using the software DIYABC 2.0.3 (Cornuet et al., 2008) based on the dataset at the genet levels (i.e., including only one representative of each MLL). Different scenarios about population history were compared and their relative support was assessed using direct and logistic regression analyses. Pools of meadows were constructed according to FCA results (central‐western, north‐western, northern, and north‐eastern geographical clusters were pooled and compared). The Cyprus sample was relatively small and thus excluded from this particular analysis. Broad log‐normal priors were used (Table 3) and multiple scenarios were tested considering alternative division times and demographics. We tested a total of 16 scenarios (Figure S1). The 16 × 10^6^ data sets were implemented for each scenario. The best fit was obtained by direct and logistic regressions of each scenario. Given the best‐supported scenario, we then assessed the probability of the deviation between simulated and observed summary statistics for the number of alleles, gene diversity, and F_ST_. According to Short et al. (2007), the generation interval of *C. nodosa* is estimated at 3 years, and based on this interval the relative times were calculated.

## 3 RESULTS

### 3.1 Clonality

Not surprisingly (given our sampling strategy which was designed to avoid replicates), nearly all of the 335 genotyped sampling units (SUs) were unique MLG. Only 3 MLLs were detected across the whole data set (Table 1). Analyses were performed for both data sets (distinct MLG and including these 3 MLLs). Expectedly given the low number of MLL, results did not differ significantly (data not shown), thus the initial data set of 335 SUs was further used.

**TABLE 1:** Summary population anthropogenic impact (AI), number of SUs (*N*), clonal (MLG, MLL), and genetic measures (H_EXP_: expected Heterozygosity; H_OBS_: observed Heterozygosity; Allelic richness, number of alleles, fixation index F_IS_) estimated for population samples of *Cymodocea nodosa* across the study area

Populations	AI	*N*	MLG	MLL	H_EXP_	H_OBS_	Allelic richness	No_alleles	F_IS_
LEM	Small	8	8		0.569	0.507	3.271	3.556	0.100
IME	High	32	32		0.716	0.543	4.233	7.056	**0.247**
FAN	High	34	34		0.749	0.637	4.796	8.333	**0.164**
VRA	Small	34	33	1	0.703	0.575	4.395	7.556	**0.215**
NKA	High	22	22		0.690	0.651	4.236	6.500	0.091
VAI	High	23	23		0.625	0.532	3.636	5.167	**0.146**
AGT	High	38	38		0.695	0.566	3.946	7.056	**0.211**
CHA	High	9	9		0.595	0.778	3.111	3.111	−0.363
EPA	Mediate	47	47		0.681	0.548	4.114	7.500	**0.221**
WPA	High	36	36		0.662	0.600	3.920	6.111	**0.134**
MAG	High	37	35	2	0.662	0.672	4.067	6.500	0.041
CYP	Small	11	11		0.602	0.582	3.378	3.833	0.000

Note

Values in bold indicate significance for adjusted nominal level (5%) for multiple comparisons (*p* < .0023).

Abbreviations: AGT, Ag. Triada; CHA, Chalkidiki; CYP, Cyprus; EPA, eastern Pagasitikos; FAN, Fanari; IME, Imeros; LEM, Lemnos; MAG, Maliakos Gulf; NKA, Nea Karvali; VIA, Viamyl; VRA, Vrasidas; WPA, western Pagasitikos.

### 3.2 Within population polymorphism

A moderately high level of polymorphism was detected in all tested loci, ranging from a few loci (1–10 loci) for the majority of populations, to 16 alleles at the Cn4‐27 locus (see Table S1). Observed heterozygosity values were quite low across all loci for all populations (overall mean H_OBS_ = 0.599) and were comparatively lower than overall expected heterozygosity values (mean H_EXP_ = 0.663). Values of H_OBS_, H_EXP_, F_IS_, number of alleles, and allelic richness are shown in Table 1.

F_ST_ values between the 12 meadows ranged from 0.0214 between Fanari (north‐eastern Aegean) and Vrasidas (north Aegean) to 0.2937 between Cyprus and Lemnos (north‐eastern Aegean). Most of the values were highly significant after correction for multiple tests (a = 0.05, *p* = .000758) as shown in Table 2.

**TABLE 2:** F_ST_ pairwise values between the twelve meadows in the eastern Mediterranean Sea

	LEM	IME	FAN	VRA	NKA	VIA	AGT	CHA	EPA	WPA	MAG
IME	0.072	0									
FAN	**0.243**	**0.177**	0								
VRA	**0.261**	**0.195**	**0.021**	0							
NKA	**0.269**	**0.200**	**0.049**	**0.048**	0						
VIA	**0.221**	**0.181**	**0.148**	**0.142**	**0.154**	0					
AGT	**0.220**	**0.155**	**0.120**	**0.104**	**0.131**	**0.057**	0				
CHA	0.255	**0.175**	**0.151**	**0.168**	**0.188**	**0.117**	**0.141**	0			
EPA	**0.217**	**0.161**	**0.105**	**0.102**	**0.124**	**0.044**	**0.061**	**0.117**	0		
WPA	**0.261**	**0.204**	**0.095**	**0.082**	**0.116**	**0.095**	**0.092**	**0.127**	**0.075**	0	
MAG	**0.212**	**0.181**	**0.126**	**0.129**	**0.156**	**0.053**	**0.092**	**0.091**	**0.060**	**0.078**	0
CYP	0.294	**0.156**	**0.222**	**0.255**	**0.277**	**0.230**	**0.187**	0.293	**0.191**	**0.267**	**0.224**

Note

Values in bold indicate significance for adjusted nominal level (5%) for multiple comparisons (*p* < .000758).

Abbreviations: AGT, Ag. Triada; CHA, Chalkidiki; CYP, Cyprus; EPA, eastern Pagasitikos; FAN, Fanari; IME, Imeros; LEM, Lemnos; MAG, Maliakos Gulf; NKA, Nea Karvali; VIA, Viamyl; VRA, Vrasidas; WPA, western Pagasitikos.

### 3.3 Population structure

**FIGURE 2:**
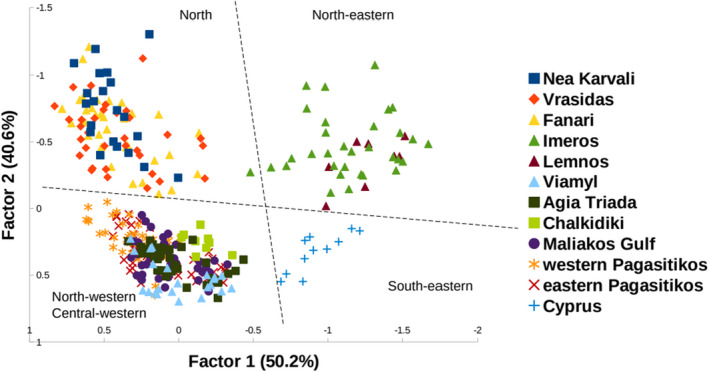
Factorial correspondence analysis (FCA) of *Cymodocea nodosa* meadows multilocus scores computed using GENETIX. Multilocus scores are computed in the bivariate space defined by the first two factorial components. Dotted lines pinpoint nominal distinctions across the east–west and north–south axes (south‐eastern: Cyprus; north‐eastern: Lemnos, Imeros; North: Nea Karvali, Vrasidas, Fanari; north‐western: Agia Triada, Viamyl, Chalkidiki; central‐western: eastern Pagasitikos, western Pagasitikos, Maliakos Gulf)

Factorial correspondence analysis showed the geographical clustering of the 12 sampled meadows (Figure 2) into 4 distinct clusters: (NE) north‐eastern Aegean, (N) North Aegean, (NW) north‐western/(CW) central‐western Aegean, and (SE) south‐eastern‐Cyprus. For the analysis in STRUCTURE, LnP(K) dropped after K = 2 and ΔK = 2 (see Figure S2). At K = 2, NE Aegean seems to be the rather distinct meadow as well as the meadow of Cyprus (see Figure S3). Interestingly, results thus highlight moderate genetic connectivity among northern (Nea Karvali, Vrasidas, and Fanari), north‐western (Viamyl, Agia Triada, and Chalkidiki), and central‐western Aegean (Maliakos Gulf, western Pagasitikos, and eastern Pagasitikos) meadows.

### 3.4 Estimates

**FIGURE 3:**
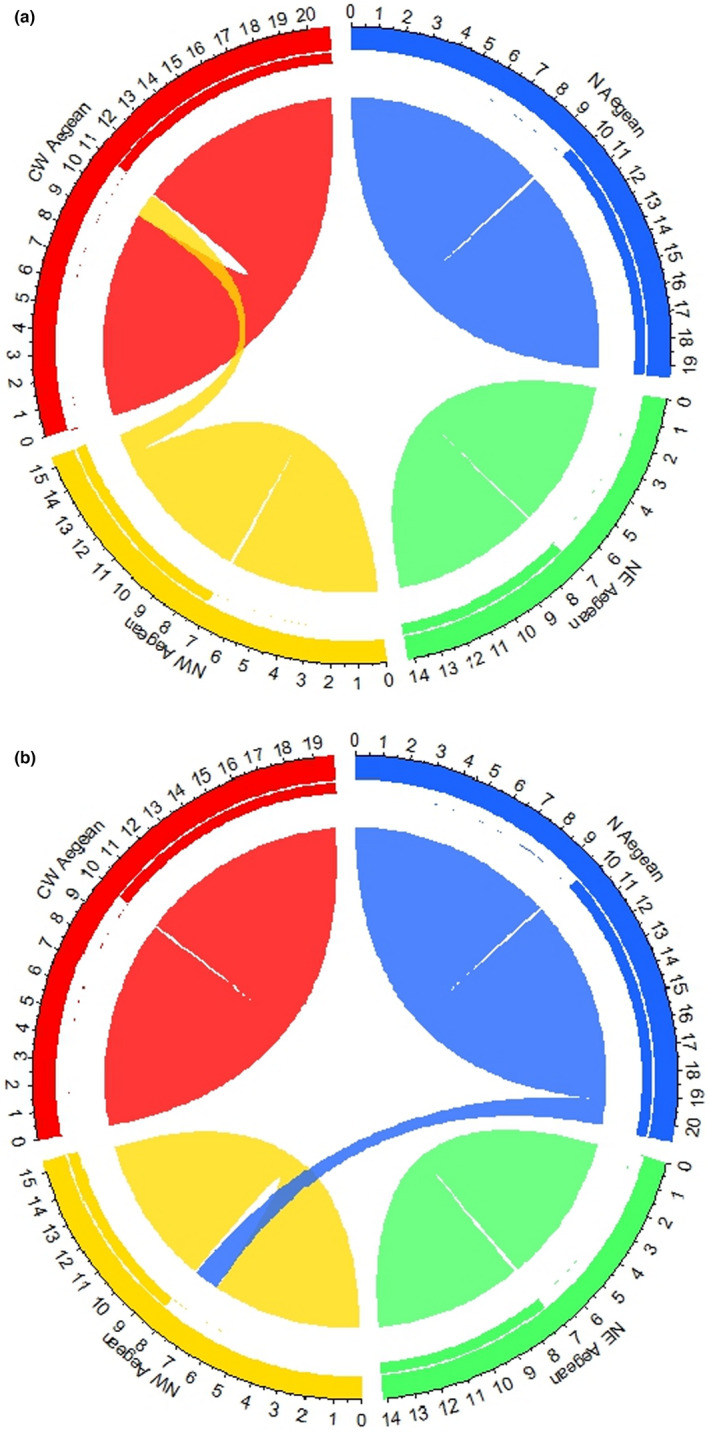
Circos plots of source‐sink migration dynamics as implemented in the R platform (v.3.2.3). Plot A corresponds to contemporary migration directionality (through BayesAss software) and B to historical migration (through Migrate software). The width of migration curves reflects the relative amount of migration. NW: north‐western; CW: central‐western; N: northern; NE: north‐eastern

The estimation of the contemporary and historical migration rates between all groups was performed using BayesAss and Migrate software, respectively (Table 3). Historical and contemporary migration analyses showed a directionality from north to north‐western and north‐western to central‐western Aegean Sea populations, respectively (Figure 3).

**TABLE 3:** (A) Contemporary migration rates through BayesAss software (SD are given in the parentheses). (B) Migrate results

	N_Aegean	NE_Aegean	NW_Aegean	CW_Aegean	Cyprus
A					
N_Aegean	—	0.015 (0.007)	0.030 (0.011)	0.010 (0.006)	0.004 (0.004)
NE_Aegean	0.008 (0.007)	—	0.008 (0.007)	0.007 (0.007)	0.009 (0.008)
NW_Aegean	0.005 (0.005)	0.005 (0.005)	—	0.114 (0.025)	0.008 (0.008)
CW_Aegean	0.004 (0.003)	0.003 (0.003)	0.0042 (0.004)	—	0.053 (0.015)
Cyprus	0.021 (0.02)	0.021 (0.02)	0.021 (0.02)	0.021 (0.02)	—
B					
N_Aegean		3.177	11.905	0.601	
NE_Aegean	2.940	—	3.194	2.261	
NW_Aegean	0.750	0.703	—	4.595	
CW_Aegean	1.596	6.339	4.354	—	

Note

(A) Values are given as m, the proportion of migrants per generation from each population on the left (row headings) to the populations on the right (column headings). (B) Historical migration rates (*M*) from each population on the left (row headings) into the populations on the right (column headings).

### 3.5 Biogeographic inferences

**FIGURE 4:**
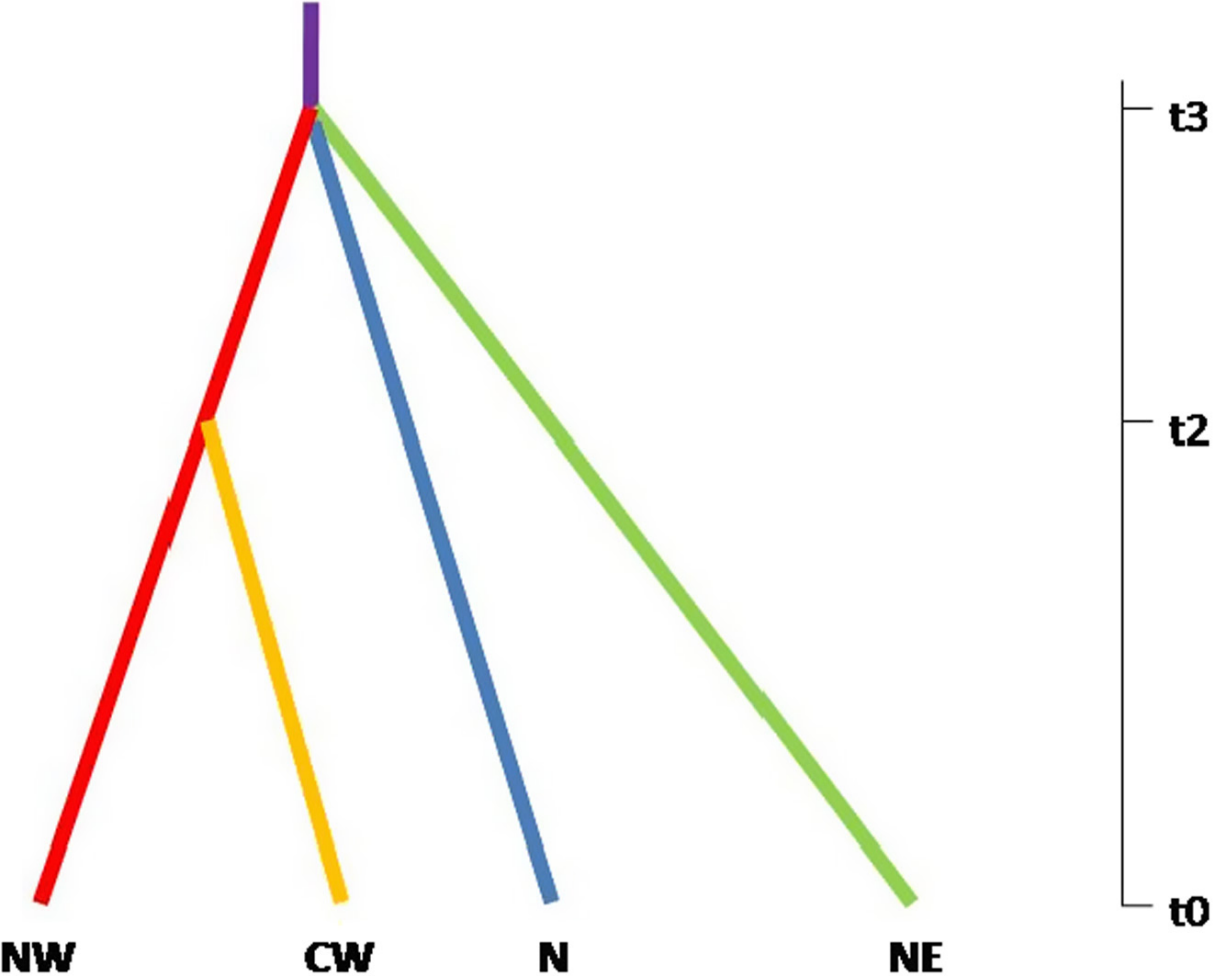
The most supported demographic scenario (scenario 13) using approximate Bayesian computation (ABC). NW: north‐western; CW: central‐western; N: northern; NE: north‐eastern. t2 is the divergent time in the Holocene epoch and t3 is the divergent time in the Pleistocene epoch (axis is not on scale)

Among the 16 different demographic scenarios examined, the most likely scenario emphasized by DIYABC analysis as seen in Figure 4 (for logistic and direct regression see Table S2; Figure S4; PCA in Figures S5 and S6) estimated that the population split in the western Aegean Sea occurred approximately 8370 years ago. This timeframe corresponds to a rather recent divergence (early Holocene period) compared to the divergence of the other three meadows (N, NE, and W Aegean Sea) which would have taken place about 103,500 years ago, during the late Pleistocene epoch (Table 4).

**TABLE 4:** DIYABC prior distribution settings and parameter estimate using 13 × 10^6^ data sets simulated under scenario 13

Parameter	Prior range	Mode	Median	95% CI low	95% CI high
Ne1 (N Aegean)	10–10^7^	2.67 × 10^4^	4.93 × 10^4^	1.37 × 10^4^	4.90 × 10^5^
Ne1 (NE Aegean)	10–10^7^	1.27 × 10^4^	2.30 × 10^4^	6.41 × 10^3^	2.58 × 10^5^
Ne3 (NW Aegean)	10–10^7^	9.15 × 10^3^	1.49 × 10^4^	4.30 × 10^3^	1.48 × 10^5^
Ne4 (CW Aegean)	10–10^7^	1.53 × 10^5^	2.44 × 10^5^	3.97 × 10^4^	2.82 × 10^6^
Na (Ancestral)	10–10^5^	10	223	17.5	2.42 × 10^3^
t0 (generations)	10–10^4^	13.1	324	16.6	4.84 × 10^3^
t2 (generations)	10–10^6^	1.53 × 10^3^	2.93 × 10^3^	476	1.73 × 10^4^
t3 (generations)	10–2 × 10^6^	9.27 × 10^3^	1.33 × 10^4^	3.74 × 10^3^	6.85 × 10^4^

Note

1. (N_e_: contemporary effective population size) 2. a: Ancestral, N: northern, NE: north‐eastern, NW: north‐western, CW: central‐western.

## 4 DISCUSSION

*Cymodocea nodosa* meadows of the eastern Mediterranean Sea exhibit a relatively high genetic differentiation. A key finding here is the existence of relatively strong barriers to dispersal between north‐eastern and other Aegean meadows, the former appearing genetically slightly closer to Cyprus meadows than to their Aegean counterparts, both through clustering and F_ST_ analysis. Such a recorded pattern is not in line with geographic distances or proximity, but is logical since a re‐colonization pattern of the north‐eastern Aegean Sea from Cyprus, gradually through the coasts of Asia Minor, has also been hypothesized for other marine plants (Orfanidis & Breeman, 1999; Parker & Breeman, 1986). One should also bear in mind that the Lemnos plateau (today's seawater depth below 100 m) during the last glacial period was united in the east with Asia Minor. *C. nodosa* population in Imeros coasts (Thrace shallow plateau) may have also been re‐colonized later on by Lemnos strain though Samothraki Island anti‐cyclone drift that also limited species westward migration. Also, perhaps the deep waters (basins) of the Chalkidiki peninsula and the Sporades islands (western and central Aegean Sea) could have served as a reservoir for preserving the species during the last glacial period (Por & Dimentman, 2006).

This may stem from historical segregation between the western and the eastern basin during the glaciation; the ancient divergence of north‐eastern meadows following historical exchange, with a lack of present‐day connectivity, is a fact that is respectively supported by our DIYABC, Migrate, and BayesAss analysis. Second, the current circulation of the north Aegean Sea may have a strong effect on gene flow and dispersal ability. Similar patterns of genetic differentiation due to cyclonic circulation have been also observed for other geographic areas and marine organisms, such as the seagrass *Thalassia hemprichii* (Jahnke et al., 2019), corals (van der Ven et al., 2016), and sponges (Voultsiadou, 2005). Third, strong local winds and the inflow of cold, low salinity waters from the Black Sea through the Dardanelles Strait (Samothraki anticyclone), as well as that from Levantine Sea waters reaching the Lemnos‐Lesvos plateau (Tzali et al., 2010) may also represent potential barriers to gene flow. The historical, current, and environmental hypotheses are not mutually exclusive but may be complementary, historical divergence being possibly presently maintained by current, wind, and inflow of waters preventing the homogenization of the two genetic clusters.

Factorial component analysis clearly formed three distinct groups in the Aegean Sea and ABC analysis revealed an initial split of the *C. nodosa* populations between the north‐western, northern, and north‐eastern Aegean during the Pleistocene epoch, followed by a more recent divergence of the north‐western population, with a new population the central‐western part of the Aegean Sea. According to our findings, the most likely historical scenario matches the divergence according to the Pleistocene epoch at first (north, north‐east vs. north‐west and central‐west), and then fits the LGM and Holocene conditions for the split between north‐western and central‐western. Moreover, historical and contemporary migration analyses showed a directionality from north to north‐western and north‐western to central‐western, respectively, in agreement with the most likely scenario supported by our coalescent scenario.

Although less differentiated among themselves, meadows from the western part of the Aegean Sea exhibit strong differentiation, yet BayesAss suggests hints of contemporary exchange between central‐western Aegean and north‐western Aegean meadows (Figure 3). Such apparently contradictory results are not necessarily surprising for seagrasses and can be reconciled considering their genetic patchiness. Seagrass meadows form mosaic patches depending on the colonization timing, resulting in systematic differentiation even for patches sampled a few meters apart, as detailed for *Zostera marina* (Becheler et al., 2010). This is an important feature of the north Aegean basin, as this observed barrier to gene flow between northern and north‐eastern Aegean basin is possibly due to an action of strong local winds, the inflow of cold, low salinity waters from the Black Sea through the Dardanelles Strait (Samothraki anticyclone), and the inflow of waters with Levantine Sea origin that reach up to the Lemnos‐Lesvos plateau (Tzali et al., 2010).

Clonal growth is a key life‐history strategy for marine angiosperms (Arnaud‐Haond et al., 2007), and studies argue that clonal reproduction could influence patterns of genetic differentiation (Coyer et al., 2004; Procaccini et al., 2002). Here, the sampling strategy defined to optimize the capture of genetic polymorphism resulted in the near absence of MLL that does not allow assessing the importance of clonal reproduction in this area. Furthermore, the significant deficit in heterozygotes is likely to result from technical issues such as null alleles that do not allow using F_IS_ as an indicator of the level of clonality (Arnaud‐Haond et al., 2020). All microsatellites were previously developed from samples of the western part of the Mediterranean Sea, which due to the phylogeographic differentiation between the two basins may indeed affect the genotyped number of alleles, resulting in low allelic richness in our study area and exhibiting significant heterozygote deficiency in many loci, contrasting with the observations on *C. nodosa* in other parts of its distribution range (Alberto et al., 2008). On the other hand, results obtained with this sampling strategy of replicate avoidance surely deliver more detailed information as to the distribution of genetic diversity and the present and past connectivity patterns in this usually underexplored Mediterranean area.

There are limited data on the population genetic structure of seagrasses in the Mediterranean Sea, in particular showing differential genetic patterns among species and areas that eventually suggest a different history and life strategy of each species (Arnaud‐Haond et al., 2020) and a peculiar distribution of a mosaic pattern of genetic differentiation at the meadow scale. This fact certainly challenges the genetic population concept (Becheler et al., 2010), and is likely due to the turn‐over of individuals in meadows mostly driven by local extirpation followed by fine‐grain recolonization of available space through dispersed propagules (Becheler et al., 2013; Gkafas et al., 2016). Understanding the historical and contemporary drivers of the distribution of genetic diversity, connectivity and turnover dynamics of meadows is critical for forming effective conservation and management policies. Interestingly, still detectable signatures of ancient events such as last glacial fluctuations of the Mediterranean Sea level support the hypothesis of either very low turn‐over or connectivity, possibly influenced by abiotic drivers such as the currents, or jets of water masses with contrasted temperature. Such information is necessary to fuel the management strategies deployed for seagrasses and progressively for other species (Conides et al., 2020) in the Mediterranean Sea.

## AUTHOR CONTRIBUTIONS

**Ioannis Konstantinidis:** Data curation (equal); Methodology (equal); Software (equal); Writing – original draft (equal). **Georgios A. Gkafas:** Data curation (equal); Investigation (equal); Methodology (equal); Software (equal); Validation (equal); Writing – original draft (equal). **Vasillis Papathanasiou:** Data curation (equal); Investigation (equal); Software (equal). **Sotiris Orfanidis:** Data curation (equal); Formal analysis (equal); Investigation (equal); Methodology (equal); Validation (equal); Writing – review & editing (equal). **Frithjof C. Küpper:** Data curation (equal); Formal analysis (equal); Investigation (equal); Validation (equal); Writing – review & editing (equal). **Sophie Arnaud‐Haond:** Data curation (equal); Formal analysis (equal); Investigation (equal); Methodology (equal); Software (equal); Validation (equal); Writing – review & editing (equal). **Athanasios Exadactylos:** Conceptualization (equal); Data curation (equal); Funding acquisition (equal); Investigation (equal); Methodology (equal); Project administration (equal); Resources (equal); Software (equal); Supervision (equal); Validation (equal); Writing – review & editing (equal).

## Supporting information

Appendix S1

## Data Availability

The dataset (gene_pop file) of this study is available upon request as Supplementary Material with a separate assigned.
