# Supplementary material for: Biogeography pattern of the marine angiosperm Cymodocea nodosa in the eastern Mediterranean Sea related to the quaternary climatic changes
Source: Ecol Evol. 2022 May 25;12(5):e8911. doi: 10.1002/ece3.8911 (PMC9131598; doi:10.1002/ece3.8911)
Supplement: Supplementary file 1 — Appendix S1 [file ECE3-12-e8911-s001.docx]

**Supplementary Information of the ms titled:**

“Biogeography pattern of the marine angiosperm *Cymodocea nodosa* in the eastern Mediterranean Sea related to the quaternary climatic changes”

**Table S1.** The panel of the 18 microsatellite markers, along with genetic indices across the twelve geographical populations of the *Cymodocea nodosa* meadows in the study area. No: number of alleles; A: allelic richness. **LEM: Lemnos, IME: Imeros, FAN: Fanari, VRA: Vrasidas, NKA: Nea Karvali; VIA: Viamyl; AGT: Ag. Triada; CHA: Chalkidiki; EPA: eastern Pagasitikos; WPA: western Pagasitikos; MAG: Maliakos Gulf; CYP: Cyprus**

| Locus | Genetic Indices | **LEM** | **IME** | **FAN** | **VRA** | **NKA** | **VIA** | **AGT** | **CHA** | **EPA** | **EPA** | **MAG** | **CYP** |
| --- | --- | --- | --- | --- | --- | --- | --- | --- | --- | --- | --- | --- | --- |
| Cn2-14 | H_EXP_ | 0.802 | 0.702 | 0.743 | 0.349 | 0.895 | 0.591 | 0.615 | 0.822 | 0.731 | 0.489 | 0.854 | 0.727 |
|  | H_OBS_ | 0.714 | 0.531 | 0.839 | 0.219 | 1.000 | 0.222 | 0.189 | 1.000 | 0.667 | 0.448 | 0.938 | 0.546 |
|  | A | 4.426 | 3.863 | 4.381 | 2.410 | 6.534 | 3.171 | 3.420 | 4.000 | 4.687 | 2.818 | 5.877 | 3.644 |
|  | No | 5 | 7 | 9 | 5 | 12 | 5 | 7 | 4 | 9 | 4 | 11 | 4 |
|  | F_IS_ | 0.118 | 0.247 | -0.131 | 0.377 | -0.121 | **0.630** | **0.695** | -0.250 | 0.088 | 0.085 | -0.099 | 0.259 |
| Cn2-16 | H_EXP_ | 0.484 | 0.670 | 0.795 | 0.821 | 0.837 | 0.559 | 0.660 | 0.000 | 0.670 | 0.774 | 0.763 | 0.610 |
|  | H_OBS_ | 0.286 | 0.656 | 0.516 | 0.594 | 1.000 | 0.444 | 0.351 | 0.000 | 0.689 | 0.862 | 0.844 | 0.818 |
|  | A | 2.868 | 3.558 | 4.631 | 4.947 | 5.215 | 3.319 | 3.533 | 1.000 | 3.811 | 4.202 | 4.210 | 2.852 |
|  | No | 3 | 5 | 6 | 7 | 7 | 5 | 6 | 1 | 7 | 5 | 6 | 3 |
|  | F_IS_ | 0.429 | 0.020 | 0.354 | 0.280 | -0.202 | 0.209 | **0.471** | NA | -0.028 | -0.116 | -0.108 | -0.364 |
| Cn2-18 | H_EXP_ | 0.769 | 0.793 | 0.846 | 0.833 | 0.615 | 0.478 | 0.666 | 0.533 | 0.591 | 0.750 | 0.800 | 0.697 |
|  | H_OBS_ | 0.857 | 0.594 | 0.710 | 0.906 | 0.421 | 0.111 | 0.460 | 0.000 | 0.333 | 0.655 | 0.844 | 0.273 |
|  | A | 4.407 | 4.607 | 5.636 | 5.316 | 3.382 | 2.710 | 3.178 | 2.000 | 3.870 | 4.460 | 4.549 | 3.722 |
|  | No | 5 | 7 | 10 | 10 | 4 | 4 | 5 | 2 | 8 | 7 | 6 | 4 |
|  | F_IS_ | -0.125 | 0.254 | 0.163 | -0.089 | 0.321 | 0.773 | 0.314 | 1.000 | **0.439** | 0.128 | -0.056 | 0.620 |
| Cn2-45 | H_EXP_ | 0.571 | 0.809 | 0.850 | 0.821 | 0.835 | 0.848 | 0.795 | 0.733 | 0.817 | 0.843 | 0.887 | 0.844 |
|  | H_OBS_ | 0.286 | 0.688 | 0.871 | 0.781 | 0.950 | 0.778 | 0.730 | 1.000 | 0.955 | 0.690 | 0.969 | 0.818 |
|  | A | 3.418 | 4.916 | 5.658 | 5.174 | 5.429 | 5.585 | 4.818 | 4.000 | 5.019 | 5.693 | 6.263 | 5.176 |
|  | No | 4 | 9 | 11 | 8 | 8 | 8 | 9 | 4 | 7 | 10 | 11 | 6 |
|  | F_IS_ | 0.520 | 0.152 | -0.025 | 0.050 | -0.142 | 0.085 | 0.083 | -0.429 | -0.170 | 0.185 | -0.093 | 0.032 |
| Cn2-86 | H_EXP_ | 0.791 | 0.726 | 0.551 | 0.586 | 0.470 | 0.572 | 0.621 | 0.689 | 0.664 | 0.635 | 0.602 | 0.723 |
|  | H_OBS_ | 0.286 | 0.194 | 0.321 | 0.172 | 0.188 | 0.235 | 0.111 | 1.000 | 0.778 | 0.655 | 0.750 | 0.455 |
|  | A | 3.931 | 3.953 | 3.711 | 3.641 | 3.119 | 3.094 | 3.326 | 3.000 | 3.636 | 3.005 | 3.063 | 3.642 |
|  | No | 4 | 5 | 7 | 7 | 6 | 4 | 9 | 3 | 7 | 4 | 4 | 4 |
|  | F_IS_ | 0.657 | **0.736** | 0.421 | **0.709** | 0.609 | 0.596 | **0.823** | -0.538 | -0.173 | -0.033 | -0.251 | 0.383 |
| Cn4-27 | H_EXP_ | 0.909 | 0.897 | 0.882 | 0.907 | 0.892 | 0.879 | 0.876 | 0.822 | 0.932 | 0.890 | 0.899 | 0.714 |
|  | H_OBS_ | 0.833 | 0.903 | 0.613 | 0.719 | 1.000 | 0.778 | 0.811 | 1.000 | 0.867 | 0.931 | 0.936 | 0.636 |
|  | A | 6.455 | 6.604 | 6.490 | 6.851 | 6.343 | 6.271 | 6.224 | 5.000 | 7.440 | 6.411 | 6.696 | 4.218 |
|  | No | 7 | 14 | 15 | 16 | 10 | 11 | 13 | 5 | 16 | 11 | 13 | 5 |
|  | F_IS_ | 0.091 | -0.007 | **0.309** | 0.210 | -0.125 | 0.119 | 0.075 | -0.250 | 0.071 | -0.047 | -0.042 | 0.114 |
| Cn4-29 | H_EXP_ | 0.264 | 0.645 | 0.875 | 0.823 | 0.800 | 0.457 | 0.802 | 0.000 | 0.558 | 0.557 | 0.092 | 0.312 |
|  | H_OBS_ | 0.286 | 0.313 | 0.613 | 0.594 | 0.450 | 0.667 | 0.865 | 0.000 | 0.111 | 0.414 | 0.031 | 0.364 |
|  | A | 1.934 | 3.275 | 5.951 | 5.228 | 4.792 | 1.992 | 5.040 | 1.000 | 2.964 | 3.281 | 1.446 | 1.932 |
|  | No | 2 | 5 | 10 | 9 | 7 | 2 | 11 | 1 | 4 | 5 | 3 | 2 |
|  | F_IS_ | -0.091 | **0.519** | **0.303** | 0.282 | 0.444 | -0.478 | -0.080 | NA | **0.803** | 0.261 | 0.663 | -0.176 |
| Cn4-35 | H_EXP_ | 0.736 | 0.788 | 0.891 | 0.782 | 0.611 | 0.767 | 0.773 | 0.733 | 0.835 | 0.808 | 0.847 | 0.610 |
|  | H_OBS_ | 0.857 | 0.677 | 0.621 | 0.586 | 0.714 | 0.722 | 0.595 | 1.000 | 0.556 | 0.690 | 0.781 | 0.818 |
|  | A | 4.846 | 4.830 | 6.377 | 5.050 | 2.951 | 4.611 | 4.830 | 4.000 | 5.438 | 5.283 | 5.426 | 3.319 |
|  | No | 6 | 8 | 12 | 8 | 4 | 7 | 10 | 4 | 10 | 9 | 8 | 4 |
|  | F_IS_ | -0.180 | 0.142 | **0.307** | 0.253 | -0.176 | 0.060 | 0.233 | -0.429 | **0.337** | 0.148 | 0.078 | -0.364 |
| Cn4-5 | H_EXP_ | 0.604 | 0.637 | 0.689 | 0.735 | 0.718 | 0.713 | 0.679 | 0.644 | 0.701 | 0.762 | 0.793 | 0.827 |
|  | H_OBS_ | 0.286 | 0.313 | 0.742 | 0.625 | 0.600 | 0.833 | 0.838 | 1.000 | 0.711 | 0.655 | 0.969 | 0.818 |
|  | A | 2.714 | 3.073 | 3.522 | 3.718 | 4.183 | 4.091 | 3.494 | 3.000 | 4.117 | 4.353 | 5.117 | 5.385 |
|  | No | 3 | 5 | 4 | 4 | 6 | 7 | 5 | 3 | 8 | 7 | 9 | 7 |
|  | F_IS_ | 0.547 | **0.514** | -0.078 | 0.151 | 0.168 | -0.175 | -0.238 | -0.667 | -0.015 | 0.142 | -0.227 | 0.011 |
| Cn4-6 | H_EXP_ | 0.648 | 0.571 | 0.658 | 0.589 | 0.240 | 0.341 | 0.618 | 0.000 | 0.509 | 0.307 | 0.600 | 0.550 |
|  | H_OBS_ | 0.286 | 0.313 | 0.452 | 0.156 | 0.053 | 0.389 | 0.378 | 0.000 | 0.500 | 0.103 | 0.719 | 0.455 |
|  | A | 3.637 | 2.713 | 3.999 | 3.066 | 1.986 | 2.382 | 3.274 | 1.000 | 3.155 | 2.309 | 2.989 | 2.827 |
|  | No | 4 | 5 | 7 | 5 | 3 | 3 | 5 | 1 | 5 | 4 | 4 | 3 |
|  | F_IS_ | 0.579 | 0.457 | 0.317 | **0.738** | 0.786 | -0.144 | 0.391 | NA | 0.017 | **0.667** | -0.202 | 0.180 |
| Cy1 | H_EXP_ | 0.736 | 0.820 | 0.806 | 0.819 | 0.831 | 0.470 | 0.781 | 0.644 | 0.747 | 0.737 | 0.255 | 0.749 |
|  | H_OBS_ | 0.714 | 0.844 | 0.742 | 0.844 | 0.500 | 0.333 | 0.703 | 1.000 | 0.556 | 0.655 | 0.188 | 0.636 |
|  | A | 3.702 | 5.101 | 4.790 | 5.071 | 5.256 | 3.199 | 4.298 | 3.000 | 4.140 | 3.998 | 2.060 | 4.361 |
|  | No | 4 | 8 | 7 | 7 | 8 | 5 | 8 | 3 | 9 | 6 | 3 | 5 |
|  | F_IS_ | 0.032 | -0.029 | 0.081 | -0.030 | 0.404 | 0.297 | 0.102 | -0.667 | 0.259 | 0.113 | 0.266 | 0.157 |
| Cy16 | H_EXP_ | 0.000 | 0.733 | 0.509 | 0.497 | 0.723 | 0.332 | 0.501 | 0.644 | 0.429 | 0.716 | 0.354 | 0.710 |
|  | H_OBS_ | 0.000 | 0.625 | 0.484 | 0.406 | 0.850 | 0.389 | 0.189 | 1.000 | 0.422 | 0.828 | 0.313 | 0.546 |
|  | A | 1.000 | 3.893 | 3.543 | 3.490 | 4.038 | 2.160 | 2.500 | 3.000 | 2.869 | 3.979 | 2.530 | 3.634 |
|  | No | 1 | 6 | 9 | 9 | 6 | 3 | 3 | 3 | 6 | 6 | 5 | 4 |
|  | F_IS_ | NA | 0.149 | 0.051 | 0.185 | -0.181 | -0.178 | **0.626** | -0.667 | 0.016 | -0.160 | 0.119 | 0.241 |
| Cy17 | H_EXP_ | 0.670 | 0.772 | 0.777 | 0.734 | 0.771 | 0.732 | 0.733 | 0.822 | 0.771 | 0.698 | 0.762 | 0.264 |
|  | H_OBS_ | 0.429 | 0.438 | 0.807 | 0.750 | 0.895 | 0.611 | 0.811 | 1.000 | 0.614 | 0.828 | 0.656 | 0.286 |
|  | A | 2.988 | 4.427 | 4.473 | 4.364 | 4.559 | 3.677 | 3.718 | 4.000 | 4.282 | 3.708 | 4.021 | 1.934 |
|  | No | 3 | 6 | 7 | 8 | 8 | 4 | 5 | 4 | 7 | 5 | 5 | 2 |
|  | F_IS_ | 0.379 | **0.437** | -0.038 | -0.023 | -0.166 | 0.169 | -0.108 | -0.250 | 0.206 | -0.189 | 0.141 | -0.091 |
| Cy3 | H_EXP_ | 0.440 | 0.203 | 0.381 | 0.205 | 0.268 | 0.737 | 0.728 | 0.778 | 0.478 | 0.197 | 0.586 | 0.312 |
|  | H_OBS_ | 0.571 | 0.188 | 0.194 | 0.125 | 0.300 | 0.667 | 0.568 | 1.000 | 0.000 | 0.103 | 0.344 | 0.364 |
|  | A | 1.999 | 1.898 | 2.456 | 1.966 | 2.033 | 3.740 | 3.695 | 4.000 | 2.198 | 1.980 | 3.163 | 1.932 |
|  | No | 2 | 4 | 3 | 4 | 3 | 4 | 4 | 4 | 3 | 5 | 4 | 2 |
|  | F_IS_ | -0.333 | 0.079 | 0.497 | 0.395 | -0.123 | 0.097 | 0.223 | -0.333 | **1.000** | 0.478 | 0.417 | -0.176 |
| Cy4 | H_EXP_ | 0.703 | 0.801 | 0.882 | 0.847 | 0.628 | 0.803 | 0.795 | 0.733 | 0.892 | 0.822 | 0.819 | 0.571 |
|  | H_OBS_ | 1.000 | 0.625 | 0.968 | 0.903 | 0.765 | 0.722 | 0.703 | 1.000 | 0.600 | 0.586 | 0.844 | 0.727 |
|  | A | 3.692 | 4.992 | 6.237 | 5.526 | 3.382 | 5.009 | 5.155 | 4.000 | 6.438 | 5.034 | 5.243 | 3.361 |
|  | No | 4 | 8 | 10 | 9 | 6 | 7 | 10 | 4 | 12 | 9 | 10 | 4 |
|  | F_IS_ | -0.474 | 0.222 | -0.100 | -0.068 | -0.227 | 0.103 | 0.117 | -0.429 | **0.330** | 0.290 | -0.031 | -0.290 |
| Po-5 | H_EXP_ | 0.758 | 0.862 | 0.847 | 0.824 | 0.800 | 0.781 | 0.589 | 0.778 | 0.726 | 0.633 | 0.757 | 0.675 |
|  | H_OBS_ | 1.000 | 0.688 | 0.871 | 0.688 | 0.700 | 0.500 | 0.703 | 1.000 | 0.600 | 0.310 | 0.813 | 0.818 |
|  | A | 3.868 | 6.079 | 5.642 | 5.117 | 4.854 | 4.870 | 3.446 | 4.000 | 4.239 | 2.971 | 4.121 | 3.394 |
|  | No | 4 | 12 | 10 | 9 | 8 | 8 | 6 | 4 | 7 | 4 | 5 | 4 |
|  | F_IS_ | -0.355 | 0.205 | -0.029 | 0.168 | 0.128 | 0.366 | -0.197 | -0.333 | 0.175 | 0.514 | -0.074 | -0.224 |
| RUMR25 | H_EXP_ | 0.000 | 0.768 | 0.781 | 0.789 | 0.797 | 0.652 | 0.740 | 0.689 | 0.670 | 0.823 | 0.683 | 0.688 |
|  | H_OBS_ | 0.000 | 0.500 | 0.581 | 0.781 | 0.889 | 0.833 | 0.973 | 1.000 | 0.864 | 0.931 | 0.906 | 0.818 |
|  | A | 1.000 | 4.653 | 4.749 | 4.612 | 4.565 | 2.935 | 4.445 | 3.000 | 3.225 | 4.912 | 3.369 | 3.617 |
|  | No | 1 | 8 | 8 | 6 | 7 | 3 | 8 | 3 | 5 | 6 | 6 | 4 |
|  | F_IS_ | NA | **0.353** | 0.259 | 0.010 | -0.119 | -0.288 | **-0.320** | -0.538 | -0.293 | -0.134 | -0.334 | -0.200 |
| RUMR4 | H_EXP_ | 0.363 | 0.686 | 0.717 | 0.691 | 0.695 | 0.541 | 0.531 | 0.644 | 0.541 | 0.482 | 0.572 | 0.247 |
|  | H_OBS_ | 0.429 | 0.688 | 0.516 | 0.500 | 0.450 | 0.333 | 0.216 | 1.000 | 0.044 | 0.448 | 0.250 | 0.273 |
|  | A | 1.989 | 3.753 | 4.075 | 3.565 | 3.621 | 2.623 | 2.628 | 3.000 | 2.517 | 2.163 | 3.057 | 1.857 |
|  | No | 2 | 5 | 5 | 5 | 4 | 3 | 3 | 3 | 5 | 3 | 4 | 2 |
|  | F_IS_ | -0.200 | -0.003 | 0.284 | 0.280 | 0.358 | 0.391 | **0.596** | -0.667 | **0.919** | 0.071 | 0.567 | -0.111 |
|  | H_EXP_ | 0.569 | 0.716 | 0.749 | 0.703 | 0.690 | 0.625 | 0.695 | 0.595 | 0.681 | 0.662 | 0.662 | 0.602 |
|  | H_OBS_ | 0.507 | 0.543 | 0.637 | 0.575 | 0.651 | 0.532 | 0.566 | 0.778 | 0.548 | 0.600 | 0.672 | 0.582 |
| overall | A | 3.271 | 4.233 | 4.796 | 4.395 | 4.236 | 3.636 | 3.946 | 3.111 | 4.114 | 3.920 | 4.067 | 3.378 |
|  | No | 3.56 | 7.06 | 8.33 | 7.56 | 6.50 | 5.17 | 7.06 | 3.11 | 7.50 | 6.11 | 6.50 | 3.83 |
|  | F_IS_ | 0.100 | 0.247 | 0.164 | 0.215 | 0.091 | 0.146 | 0.211 | -0.363 | 0.221 | 0.134 | 0.041 | 0.000 |

**Table S2**. Direct and logistic regression of the 13 analyzed demographic scenarios. CI: confidence interval

| **A** |  | **S1** | **S2** | **S3** | **S4** | **S5** | **S6** | **S7** | **S8** | **S9** | **S10** | **S11** | **S12** | **S13** |
| --- | --- | --- | --- | --- | --- | --- | --- | --- | --- | --- | --- | --- | --- | --- |
| Direct Regression | Regression value | 0,018 | 0 | 0,782 | 0,124 | 0 | 0,014 | 0 | 0,002 | 0,008 | 0 | 0,052 | 0 | 0 |
|  | 95% CI low | 0 | 0 | 0.420 | 0 | 0 | 0 | 0 | 0 | 0 | 0 | 0 | 0 | 0 |
|  | 95% CI high | 0.135 | 0 | 1 | 0.413 | 0 | 0.117 | 0 | 0.041 | 0.086 | 0 | 0.246 | 0 | 0 |
|  |  |  |  |  |  |  |  |  |  |  |  |  |  |  |
| Logistic Regression | Regression value | 0,002 | 0 | 0,863 | 0,128 | 0 | 0 | 0 | 0 | 0,002 | 0 | 0,004 | 0 | 0 |
|  | 95% CI low | 0 | 0 | 0.848 | 0.113 | 0 | 0 | 0 | 0 | 0 | 0 | 0.001 | 0 | 0 |
|  | 95% CI high | 0.004 | 0.002 | 0.879 | 0.144 | 0.002 | 0.002 | 0.002 | 0.002 | 0.004 | 0.002 | 0.006 | 0.002 | 0 |

**Figure S1.** 16 Demographic scenarios as implemented in the DYI-ABC software. Pop1: northeastern, Pop2: northern, Pop3: northwestern Pop4: central-western. t are the different tested generation times of divergence. Scenarios 14, 15 and 16 detect the presence of bottleneck events.


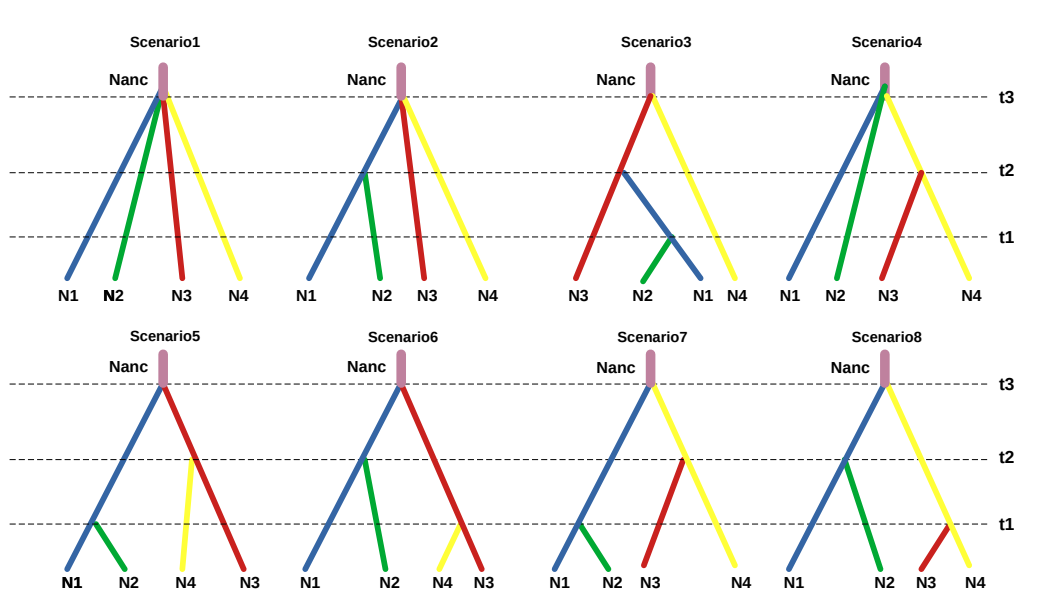


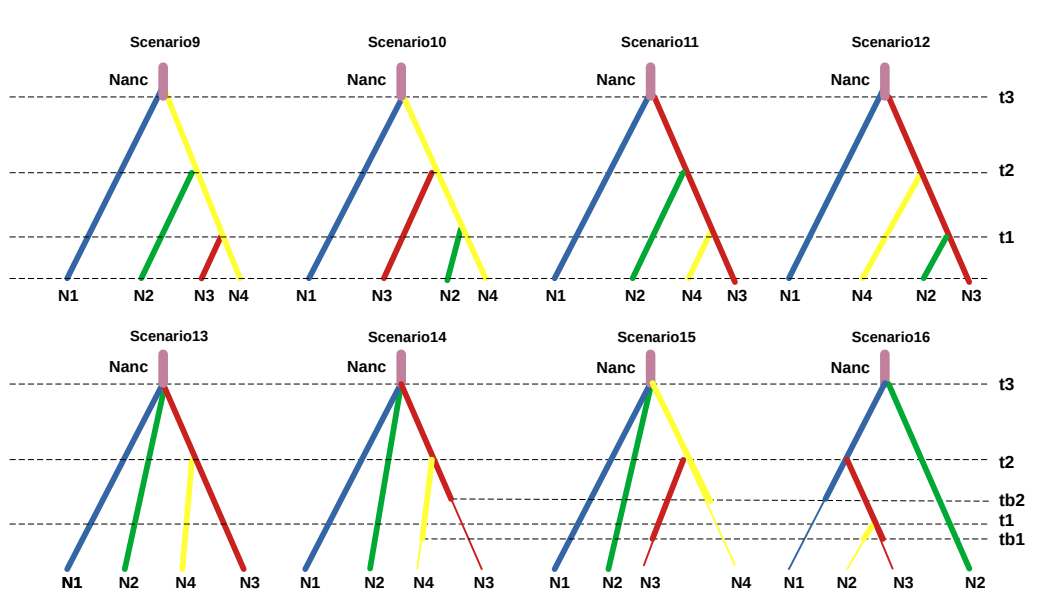


**Figure S2.** Delta K and Ln against K for Structure run given the reduction of Ln from *K* = 2 for all *Cymodocea nodosa* as implemented in HARVESTER software.


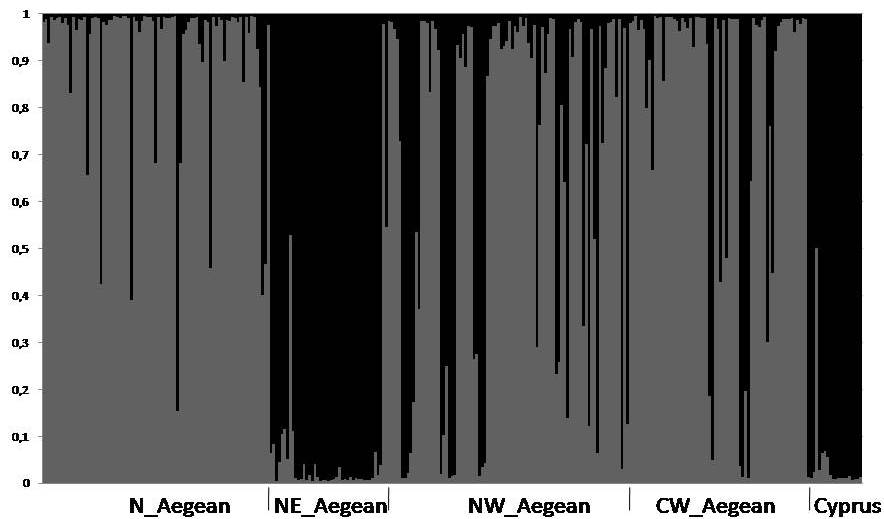


**Figure S3**. Estimated population structure plots based on genotypes of *Cymodocea nodosa* individuals. Each horizontal line represents a single individual divided into grey scale segments according to each station of membership (K = 2).


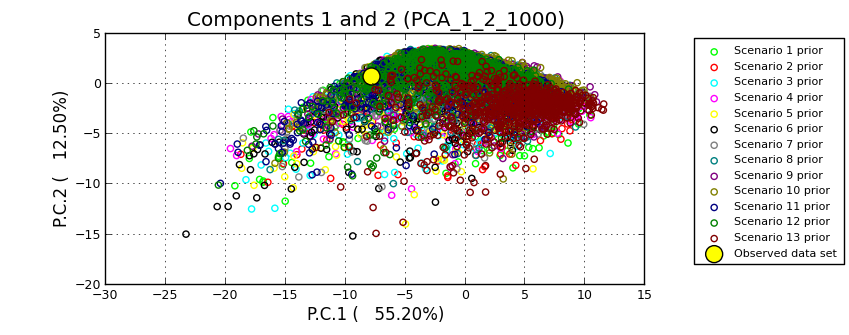


**Figure S4**: PCA plot displaying the fit between the 16scenarios simulated with uniform priors and the observed data. Plot made using DIY-ABC software.

A B


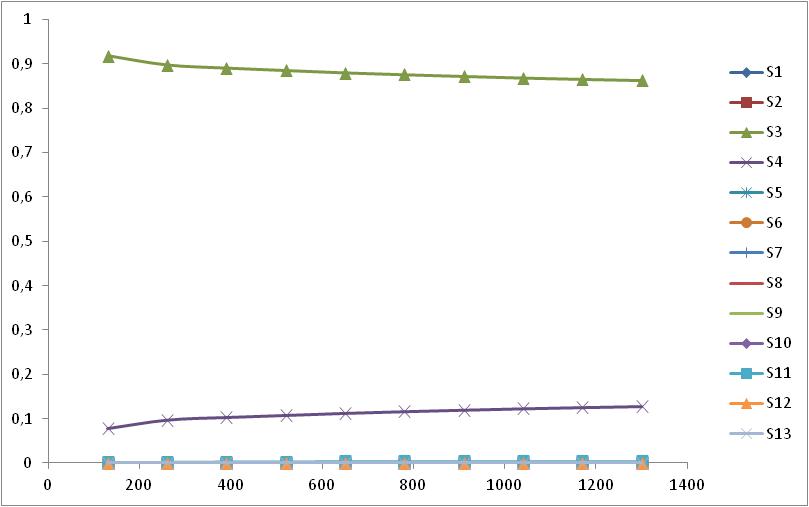

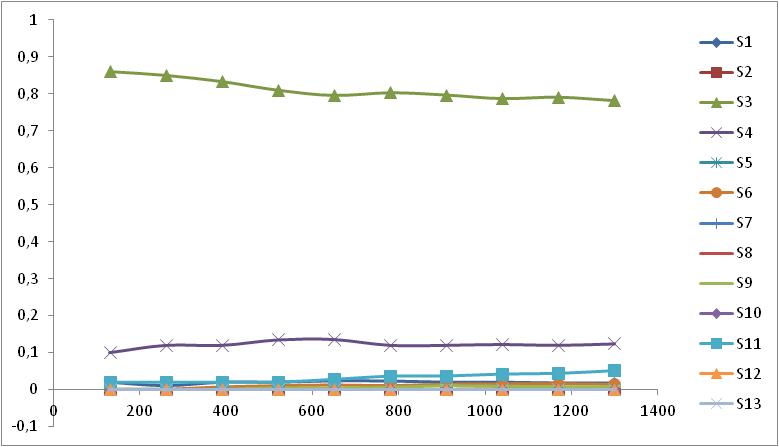


C D


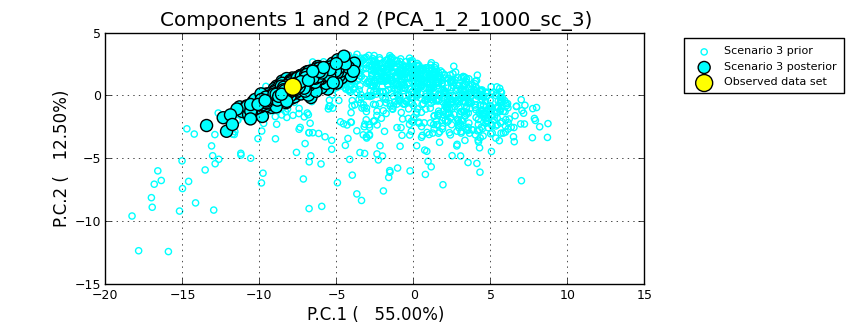


**Figure S5**. Logistic regression plot (A) and Direct regression plot (B) made in DIY-ABC to test which simulated scenarios better fit the observed data. Posterior probability in the y-axis represent the proportion of x datasets closest to the observed data that were simulated under different scenarios. (Top green line corresponds to Scenario 3). Model checking plot (D) displaying the fit of scenario 3 between prior and posterior simulated data. Plots constructed using DIYABC (Cornuet *et al*., 2008).


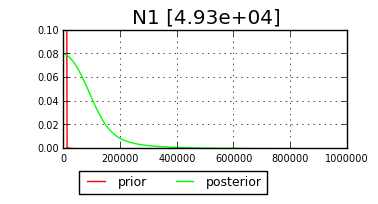

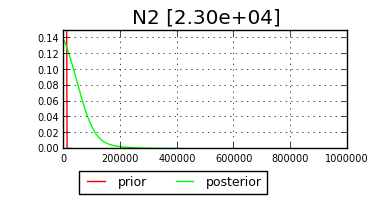


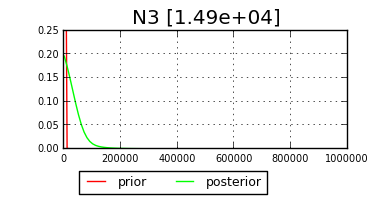

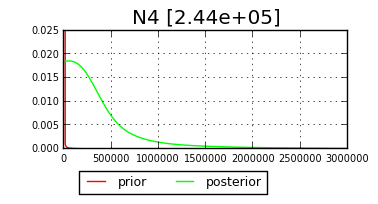


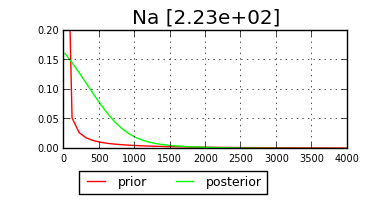

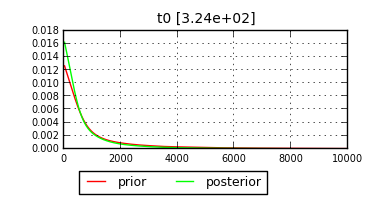


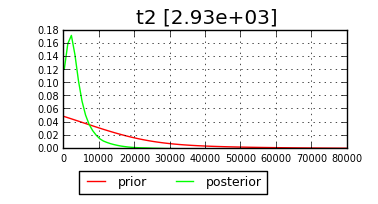

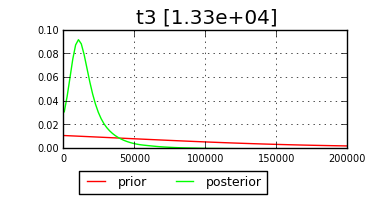


**Figure S6**: Posterior (green curve) and prior (red curve) distribution plots of ABC analysis based on 13x10^6^ simulated data sets of historical effective population sizes and time of divergence of the 3rd scenario; *N_1_*: northern Aegean, *N_2_*: north-eastern Aegean, *N_3_*: north-western Aegean, *N_4_*: central-western Aegean, *N_a_*: ancestral population, *t2*: time of split within western Aegean Sea (north-western Aegean and central-western Aegean), *t3*: time of divergence of northern Aegean, north eastern Aegean and north-western Aegean Sea.
